# *Spartina alterniflora* invasion significantly alters the assembly and structure of soil bacterial communities in the Yellow River Delta

**DOI:** 10.3389/fmicb.2025.1525632

**Published:** 2025-02-12

**Authors:** Pengyuan Sun, Yuxin Wu, Pengcheng Zhu, Jingfeng Wang, Xiaona Yu, Weihua Guo

**Affiliations:** ^1^Qingdao Key Laboratory of Ecological Protection and Restoration, Ministry of Natural Resources Key Laboratory of Ecological Prewarning, Protection and Restoration of Bohai Sea, School of Life Sciences, Shandong University, Qingdao, China; ^2^State Key Laboratory of Water Environment Simulation, School of Environment, Beijing Normal University, Beijing, China

**Keywords:** assembly process, bacterial community, coastal wetlands, co-occurrence network, *Spartina alterniflora* invasion

## Abstract

Soil microbial communities are integral to almost all terrestrial biogeochemical cycles, which are essential to coastal wetland functioning. However, how soil bacterial community assembly, composition, and structure respond to native and non-native plant invasions in coastal wetlands remains unclear. In this study of the coastal wetlands of the Yellow River Delta in China, the assembly, community composition, and diversity of soil bacterial communities associated with four wetland plant species (*Phragmites australis*, *Spartina alterniflora*, *Suaeda salsa*, and *Tamarix chinensis*) and four soil depths (0–10 cm, 10–20 cm, 20–30 cm, and 30–40 cm) were characterized using high-throughput sequencing. Plant species identity, as well as environmental factors, rather than soil depth, was found to play predominant roles in shaping the diversity and structure of wetland soil bacterial communities. *S. alterniflora* invasion altered bacterial community structure and increased bacterial diversity. *Phragmites australis*-associated bacterial communities were enriched with sulfate-reducing bacteria such as *Desulfurivibrio* and *Desulfuromonas*. In comparison, *S. alterniflora*-associated bacterial communities were enriched with both sulfate-reducing bacteria (*SEEP-SRB1*) and sulfate-oxidizing bacteria (*Sulfurimonas*), which maintained a dynamic balance in the local sulfur-cycle, and thereby enhanced *S. alterniflora* growth. In addition, stochastic processes dominated the assembly of soil bacterial communities associated with all four plant species, but were most important for the *S. alterniflora* community. The *S. alterniflora*-associated bacterial community also showed stronger interactions and more extensive connections among bacterial taxa; a co-occurrence network for this community had the greatest average clustering coefficient, average degree, modularity, and number of links and nodes, but the lowest average path length. Altogether, individual plant species had distinct effects on soil bacterial community assembly and structure, with the invasive species having the strongest impact. These results provide insights into microbial ecology and inform management strategies for coastal wetland restoration.

## 1 Introduction

Wetlands are among the most sensitive ecosystems to global climate change. Despite covering only 5–8% of the global land area, wetlands store 20–30% of all soil carbon (C) in terrestrial ecosystems (Tan et al., 2020; Wang et al., 2019). As dynamic zones bridging terrestrial and marine environments, coastal wetlands are critical for biodiversity maintenance, biogeochemical cycling, and climate regulation (Li et al., 2024; Osland et al., 2022). However, land-use changes and sea-level rise over the past few decades have resulted in the loss of large areas of coastal wetlands globally, severely weakening their capacity to provide ecosystem services (Osland et al., 2022; Wang et al., 2019). Soil microbial communities are key drivers of biogeochemical cycles in terrestrial ecosystems, and understanding their responses to environmental changes is crucial for maintaining the functioning and stability of coastal wetlands (Zhou et al., 2020).

Soil bacterial communities are highly sensitive to environmental change (Hermans et al., 2020) and are often shaped by the local plant community, with shifts in the abundance of specific taxa explaining the “home-field advantage” effect on litter decomposition (Philippot et al., 2013). Plants directly affect soil microbes via the provision of rhizodeposits and leaf litter (Cline and Zak, 2015; Philippot et al., 2013), as well as indirectly by altering the rhizosphere environment (Zhalnina et al., 2018). In coastal wetlands, native plant communities are threatened by invasive plant species and significant losses of native plant diversity (Zhang G. et al., 2021). These changes in wetland plant community composition may greatly alter the soil microenvironment, for example by affecting nutrient availability and the soil pH (Sardans et al., 2017; Zhang et al., 2019), which then influences soil bacterial community composition and structure. In addition, plant community effects on soil bacterial communities may vary with soil depth due to variability in the distribution of plant roots, thereby affecting the amount of plant litter and rhizodeposits across soil layers (Leewis et al., 2022). Variation in oxygen availability across soil layers further shapes bacterial community composition (Tian et al., 2022). Therefore, investigating how bacterial communities respond to changes in the vegetation type and soil depth is crucial for preserving the ecological functions of wetlands.

Community-assembly studies help explain species coexistence and the maintenance of diversity (Zhou and Ning, 2017). Microbial community assembly may be influenced by both deterministic and stochastic ecological processes (Zhou et al., 2014; Chen et al., 2019). Deterministic processes include environmental selection (i.e., habitat filtering) and species interactions such as antagonism and competition (Webb et al., 2002). Stochastic processes include random birth-death events, probabilistic dispersal, and unpredictable disturbances (Chase and Myers, 2011; Zhou and Ning, 2017). Soil bacterial community assembly has been widely studied, but study findings are not currently conclusive, with both deterministic (Gao et al., 2021; Huang et al., 2022; Zhang et al., 2022) and stochastic assembly processes (Hussain et al., 2023; Yue et al., 2022; Zhang et al., 2023) identified in coastal wetlands. Other factors, including diverse soil physical and chemical properties, may also impact bacterial community assembly (Huo et al., 2023; Ni et al., 2021). Additionally, changes in plant species composition can affect soil processes and thereby regulate the composition and diversity of soil microbial community composition and diversity directly or indirectly (Ward et al., 2015; Shigyo et al., 2019). The relative contribution of deterministic versus stochastic processes may differ among bacterial communities varying in diversity, with deterministic processes tending to dominate in less diverse communities (Xun et al., 2019); therefore, changes in plant species composition may alter bacterial community assembly. Further investigation is needed to assess how soil bacterial community assembly responds to changes in plant species composition.

The Yellow River Delta (YRD) is a biodiversity hotspot in China that contains extensive wetland ecosystems (Jiang et al., 2022). The YRD encompasses diverse vegetation types, with a natural successional gradient from low-to-high tide zones; wetland plant species include *Phragmites australis*, *Suaeda salsa*, and *Tamarix chinensis*. However, due to climate change and human activities, the YRD is under threat from agricultural land reclamation, oil pollution, and non-native plant invasions, all of which put local biodiversity at risk (Yu et al., 2015; Gao et al., 2016). Since its introduction to the YRD in 1990, the rapid expansion of *Spartina alterniflora* has reduced the habitat available for native plant species, thereby threatening local biodiversity (Ren et al., 2019). Therefore, the *S. alterniflora* invasion, in addition to the natural successional gradient for native plants in the YRD, provides a good opportunity to examine differences in the assembly and structure of soil bacterial communities.

In this study, four sites containing different plant species (*P. australis*, *S. salsa*, *T. chinensis*, and the invasive *S. alterniflora*) in the coastal wetlands of YRD were selected to investigate how plant species composition and soil depth (0–10 cm, 10–20 cm, 20–30 cm, and 30–40 cm) affect soil bacterial community assembly and structure. For each site, the soil physicochemical properties were analyzed, high-throughput sequencing was performed for soil bacterial DNA, and a soil bacteria co-occurrence network was constructed. The study asked: (1) Which has a stronger influence on soil bacterial community structure, plant species identity or soil depth? and (2) Does the soil bacterial community associated with *S. alterniflora* differ from that of native plant species?

## 2 Materials and methods

### 2.1 Site description and soil collection

**Figure 1:**
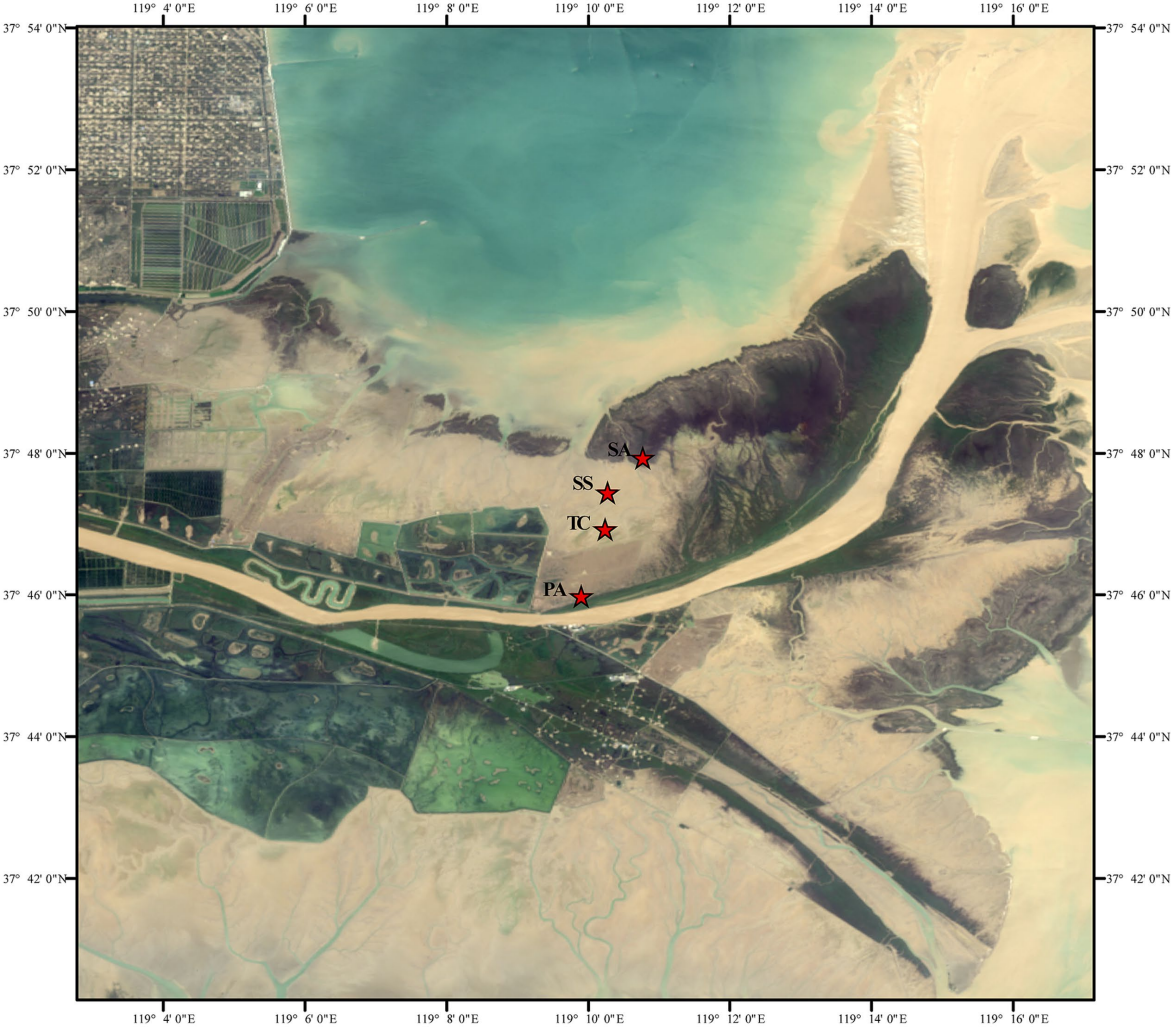
Maps of the study location and sampling sites. “PA,” “TC,” “SS,” and “SA” represent the sampling sites for *Phragmites australis*, *Tamarix chinensis*, *Suaeda salsa*, and *Spartina alterniflora*, respectively.

This study was conducted in the Yellow River Delta (YRD: 118° 33′ E–119° 20′ E, 37° 35′ N–38° 12′ N), located in Shandong Province, China. The YRD experiences a warm temperate continental monsoon climate with four seasons; the mean annual temperature ranges from 11.5–12.4°C, and the average annual precipitation from 530 to 630 mm (Lu et al., 2022). The study area represented a typical salt marsh. Four sampling sites were established in the salt marsh wetland (Figure 1), varying in their successional stage and consisting of either *S. salsa* (early successional stage, hereinafter referred to as SS), *T. chinensis* (mid-successional stage, TC), *P. australis* (late successional stage, PA), or *S. alterniflora* (invasive species, SA) (Zhang et al., 2007). These vegetation types are distributed along a sea-to-land gradient. *S. alterniflora* is closest to the sea and experiences tidal flooding during every high tide, whereas *S. salsa* and *T. chinensis* are in transitional zones with less frequent flooding. *P. australis*, located farthest from the sea near the Yellow River bank, is rarely affected by tidal flooding. Soil salinity, inferred from electrical conductivity (EC) (Liu et al., 2023), decreases along this gradient. *Spartina alterniflora* shows lower salinity due to frequent seawater flushing, while *P. australis* exhibits the lowest overall salinity near the freshwater source. This spatial and environmental gradient provides a unique natural laboratory for studying the ecological and biogeochemical processes in coastal wetlands.

Soil samples were collected from six plots (1 m × 1 m) within each site in September 2020; these were sectioned into four layers (representing soil depths of 0–10 cm, 10–20 cm, 20–30 cm, and 30–40 cm). Using a five-point sampling method, five subsamples were obtained per plot at each soil depth, and these were then thoroughly mixed to create a single representative sample. To avoid contamination with plant fragments, we first carefully removed plant litter from the soil surface before sampling. After the samples were brought back to the laboratory, any visible plant fragments were manually removed before further analysis. In total, 96 soil samples (four sampling sites × four soil depths × six replicates [plots]) were obtained and transported to the laboratory on ice. Each soil sample was divided into two parts: one was air-dried for later soil physicochemical analysis, and the second was frozen (−80°C) prior to microbial DNA extraction and high-throughput sequencing.

### 2.2 Soil physicochemical property analysis

The air-dried soil samples were sieved through 2 mm mesh to remove visible plant roots and pebbles. Soil samples were then oven-dried at 105°C to constant weight, after which they were weighed to determine the soil water content (SWC). Distilled water soil extracts (5:1 volume: weight) were used to measure the soil pH (FiveEasy Plus™, Mettler Toledo, Switzerland) and electrical conductivity (EC, FiveEasy Plus™, Mettler Toledo, Switzerland). Soil organic carbon (SOC) was determined via potassium dichromate oxidation with external heating (Nelson and Sommers, 1982). Soil dissolved organic carbon (DOC) was determined using a TOC analyzer (TOC-L CPH, Shimadzu, China) after extracting fresh soil samples with 2 mol L^−1^ KCl. Soil total nitrogen (TN) and total phosphorus (TP) were determined by the Kjeldahl method and molybdenum antimony colorimetric method, respectively. The soil nitrate nitrogen (NO_3_^−^) and ammonium nitrogen (NH_4_^+^) were quantified via a spectrophotometry-based colorimetric method after leaching samples in a 2 mol L^−1^ KCl solution.

### 2.3 DNA extraction and high-throughput sequencing

For microbial analysis, the soil was first passed through a 40-mesh (0.5 mm) sieve to remove finer plant debris, and then the DNA of soil microorganisms was extracted using a commercial DNA extraction kit (MagPure Soil DNA LQ Kit, Guangzhou Magen Biotechnology Co., Ltd.). The quality of the extracted DNA was assessed using a NanoDrop^®^ ND-1000 spectrophotometer (Thermo Fisher Scientific Inc., Waltham, MA, United States) and 0.8% agarose gel electrophoresis. The primers 343F-5’-TACGGRAGGCAGCAG-3′ and 798R-5′-AGGGTA TCTAA TCCT-3′ were used to amplify the non-conserved region (16S V3–V4) of the 16S rRNA gene (Nossa et al., 2010). To ensure the quality of the downstream analysis, raw data obtained from Illumina MiSeq sequencing were processed to remove ambiguous bases in paired-end reads. Low-quality sequences with an average quality score of less than 20 and sequences of <50 bp were also removed. Next, quality-filtered reads were merged to obtain complete paired-end sequences with a maximum overlap of 200 bp. QIIME (version 1.8.0) and UCHIME (version 2.4.2) were used to remove sequences containing N-bases, reads with single base repetitions of more than eight, reads shorter than 200 bp, and reads containing chimeric sequences. Clean reads were clustered into OTUs using Vsearch (version 2.4.2) (Rognes et al., 2016) with a 97% similarity cutoff. The most abundant sequence for each OTU was selected as the representative sequence for that OTU. The Ribosomal Database Project Classifier, a naive Bayesian classification algorithm (Wang et al., 2007), was used to compare the representative sequences and annotate these via the Silva database (version 123) (Quast et al., 2012).

### 2.4 Network analysis and visualization

Microbial networks were constructed for each study site using the relative abundance of each bacterial genus (*n* = 24). Genera that were detected in more than 18 out of 24 samples and that had a relative abundance >0.01% were included in the network analysis. Connections between genera were evaluated using Spearman rank correlation tests as implemented in the R (version 4.2.1) package “Hmisc”. Significantly-related genera (*r* > 0.7, *p* < 0.05) were retained for network construction. Network topological properties, including the average path length, clustering coefficient, density, diameter, and modularity, were calculated in Gephi (version 0.9.3), which was also used for visualization of the co-occurrence networks. A random network analysis was performed using the R package “igraph”.

### 2.5 Statistical analysis

All statistical tests were considered significant at *p* < 0.05. Differences in bacterial community *α*-diversity were assessed using one-way analyses of variance (ANOVAs) in SPSS 26.0 Statistics (SPSS, Chicago, IL, United States). A principal coordinate analysis (PCoA) based on Bray-Curtis distances was performed using the “vegdist” function in the “vegan” package in R (version 4.2.1) to visualize differences in soil bacterial community composition among study sites. To determine which bacterial taxa varied significantly in abundance among sites, a linear discriminant analysis (LDA) effect size (LEfSe) analysis was run online.^1^ Pearson correlation coefficients were used to characterize how bacterial α-diversity was related to soil physicochemical properties, while Mantel tests were implemented in the “linkET” package in R to assess how soil physicochemical properties shaped bacterial community composition. The neutral model proposed by Sloan et al. (2006) was used to assess how stochastic processes affected bacterial community assembly. Spearman’s rank correlation was used to assess the relationships between the top 30 genera in all soil bacterial communities and various soil physicochemical properties across the four study sites, as shown in Supplementary Figure S2.

## 3 Results

### 3.1 Soil physicochemical properties

At a given soil depth, soil DOC, EC, NO_3_^−^, pH, SOC, and TN varied significantly among sites (*p* < 0.05, Supplementary Table S1). The soil pH, DOC, and TN were higher in PA than the other three sites, while the soil C/N and EC were lowest in PA. In addition, the soil DOC content was higher in PA and SA versus the other study sites. Compared to other sites, the soil NO_3_^−^ content was the highest and the NH_4_^+^ content the lowest in SA. Within given site, differences in soil physicochemical properties were relatively small across soil depths (Supplementary Table S1). Several factors decreased with soil depth, including soil DOC in SS; SOC in TC; NH_4_^+^, SOC, and TN in PA; and NH_4_^+^ in SA.

### 3.2 Bacterial community diversity

**Figure 2:**
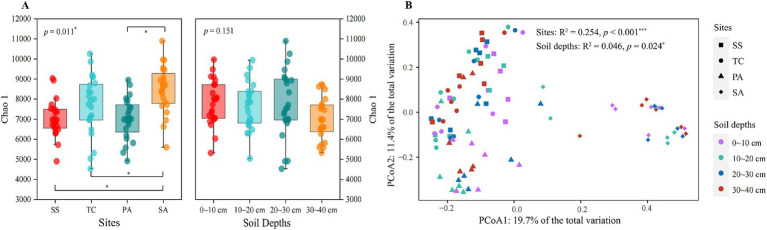
Chao 1 index (*α*-diversity) for bacterial communities from four sampling sites and soil depths **(A)**. Lines between pairs of boxes indicate significant differences (*p* < 0.05) between sites (left) or soil depths (right). A principal coordinate analysis (PCoA) was performed for bacterial community composition (based on Bray-Curtis distances) to illustrate variation among sites and soil depths **(B)**. SS, *Suaeda salsa*; TC, *Tamarix chinensis*; PA, *Phragmites australis*; SA, *Spartina alterniflora.* *p < 0.05.

A total of 33,158–66,617 high quality sequences were obtained after quality control and chimera removal, with the number of OTUs ranging from 3,608 to 8,134 per sample. Study site had a significant effect on the Chao1 index of the soil microbial community (*p* < 0.05), but no effect of soil depth was observed (*p* > 0.05). Specifically, SA had the highest Chao1 index at 8,505.75 ± 1,169.08, which was significantly higher than that of the other three sites (*p* < 0.05) (Figure 2A). However, the differences among PA, SS, and TC in the Chao1 index were not significant (*p* > 0.05). In contrast to study site, soil depth did not affect the Chao1 index (*p* > 0.05) (Figure 2A).

To assess how study site and soil depth affected bacterial community *β*–diversity, a principal coordinate analysis (PCoA) was performed based on Bray-Curtis distances. Study site had a stronger effect on bacterial community composition than soil depth (ANOVA, Site: *R*^2^ = 0.254, *p* < 0.001; Soil depth: *R*^2^ = 0.046, *p* = 0.024). The bacterial communities from SA differed most significantly from those at the other three sites. In addition, the bacterial communities from PA were moderately different from those at SS and TC (Figure 2B).

### 3.3 Bacterial community composition

**Figure 3:**
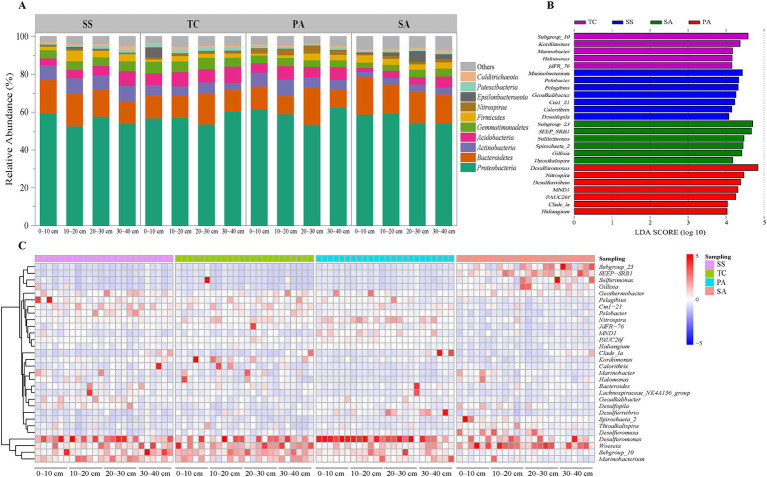
Bacterial community composition across study sites and soil depths. Relative abundance of the top ten most common bacterial phyla across sampling sites and soil depths **(A)**. Most abundant bacterial genera at each sampling site **(B)**; Heatmap of bacterial genera across sampling sites for the top 30 most abundant genera **(C)**. SS, *Suaeda salsa*; TC, *Tamarix chinensis*; PA, *Phragmites australis*; SA, *Spartina alterniflora.*

Taxonomic assignment of representative sequences for each OTU revealed a total of 59 phyla, of which the Proteobacteria (13.4–71.1%), Bacteroidetes (4.7–66.6%), Acidobacteria (1.2–13.7%), and Actinobacteria (1.8–13.8%) were the dominant phyla, together accounting for more than 80% of all reads across samples (Figure 3A). As expected, the effect of study site was stronger than that of soil depth for the Actinobacteria, Gemmatimonadetes, Nitrospirae, and Patescibacteria (*p* < 0.05; Supplementary Table S2). Notably, TC contained more Acidobacteria, Gemmatimonadetes, and Patescibacteria than the other sites (*p* < 0.05), while SS had a higher proportion of Actinobacteria (*p* < 0.05) and PA a greater relative abundance of Nitrospirae (*p* < 0.05). Finally, Acidobacteria and Calditrichaeota were significantly more abundant in the 30–40 cm soil layer (*p* < 0.05).

A total of 1,694 genera were identified from the soil samples, and a heat map was created for the 30 most abundant genera (Figure 3C). The five most common genera across all samples were *Desulfuromonas*, *Woeseia*, *Subgroup*_*10*, *Marinobacterium*, and *Nitrospira*. However, bacterial genera showed differences in abundance among the four study sites (Figure 3C). The LEfSe analysis indicated that seven genera were biomarkers for SS (Figure 3B), with *Marinobacterium*, *Pelobacter*, and *Pelagibius* the most important of these. *Subgroup_10*, *Kordiimonas*, *Marinobacter*, *Halomoans*, and *jdFR_76* were identified as biomarkers for TC, whereas six genera emerged as biomarkers for SA, including *Subgroup_23, Gillisia*, *SEEP-SRB1*, and *Sulfurimonas*. In addition, *Desulfurivibrio*, *Nitrospira*, *Desulfuromonas*, and *Desulfurivibrio* were the dominant genera at PA, while the relative abundance of *Calorithrix* was relatively low.

### 3.4 Assembly of the bacterial community

**Figure 4:**
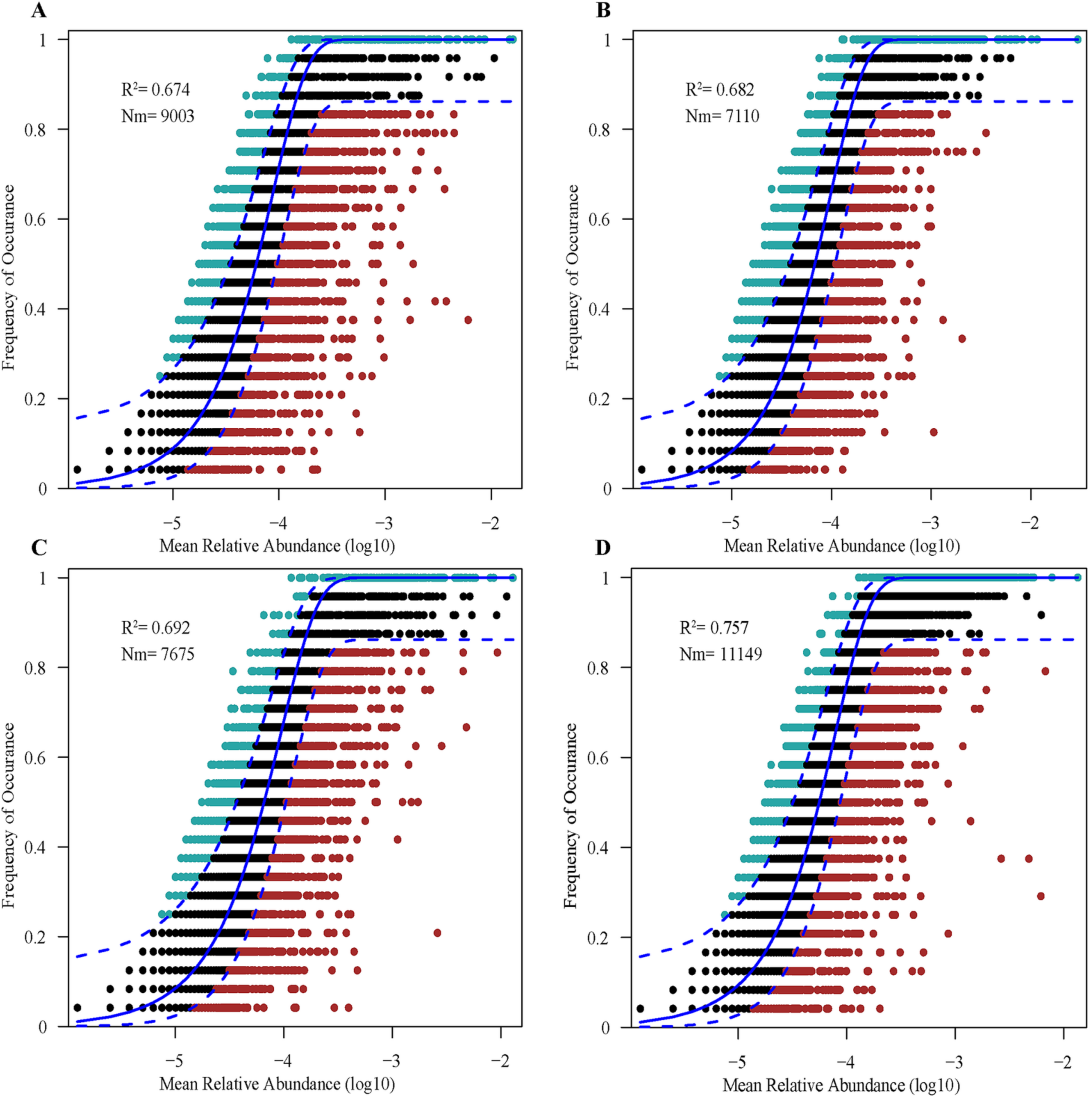
The predicted frequency of bacterial OTUs based on a neutral community model for SS **(A)**, TC **(B)**, PA **(C)**, and SA **(D)**. Solid blue lines indicate the best fit to the neutral model, and dashed blue lines represent 95% confidence intervals around model predictions. *R*^2^ denotes the coefficient of determination (a measure of model fit), and Nm represents the product of metacommunity size and the migration rate. SS, *Suaeda salsa*; TC, *Tamarix chinensis*; PA, *Phragmites australis*; SA, *Spartina alterniflora.*

The soil bacterial communities at each study site were fit to a neutral community model. The model goodness-of-fit was better for SA (explaining 75.70% of the variation in composition) than the other three sites (Figure 4). The *R*^2^ of the null models at all four sites was greater than 0.50, indicating that stochastic processes strongly shaped bacterial community assembly in the coastal wetlands of the YRD. Moreover, the migration rate was higher for bacterial communities in SA (Nm = 11,149) than other sites (Figure 4D), indicating that bacterial genera in SA were less limited by dispersal.

### 3.5 Co-occurrence networks for the bacterial communities

**Figure 5:**
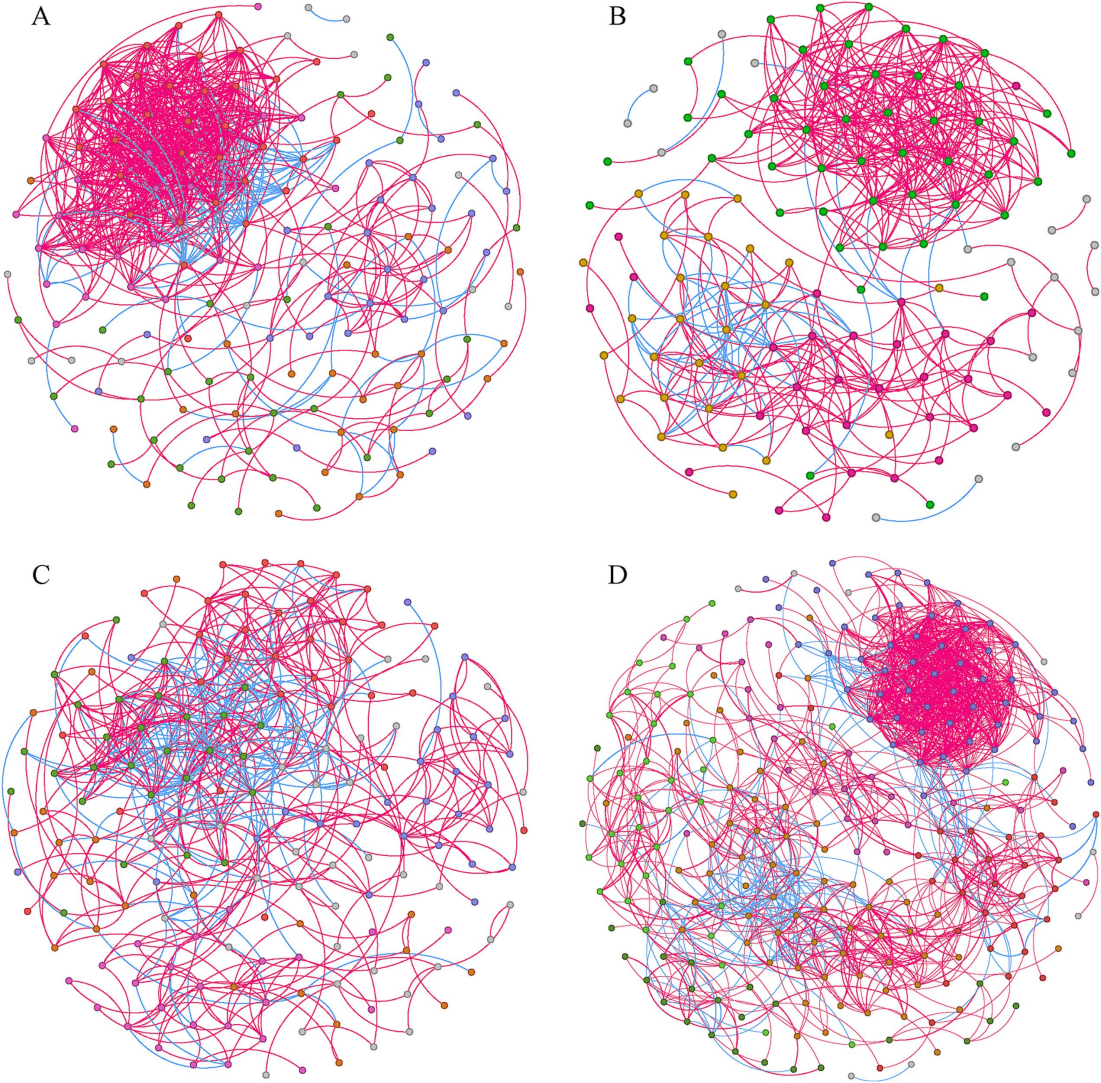
Co-occurrence networks for bacterial communities from SS **(A)**, TC **(B)**, PA **(C)**, and SA **(D)** (*n* = 24). Different modules are shown in different colors. Lines connecting two nodes (edges) represent strong (r ≥0.7) and significant correlations (*p* < 0.05). Further details on network topology are provided in Table 1. SS, *Suaeda salsa*; TC, *Tamarix chinensis*; PA, *Phragmites australis*; SA, *Spartina alterniflora.*

Four co-occurrence networks were constructed with genera plotted as nodes and the correlations between genera plotted as links (Figure 5). Overall, the number of positive links (74.27–88.94%) exceeded that of negative links (11.06–25.73%), indicating a preference for co-existence rather than co-exclusion among bacterial genera (Table 1). The co-occurrence network for SA had the greatest number of nodes (242) and links (1,287), and highest average degree (10.562), modularity (0.606), and average clustering coefficient (0.541), but the lowest average path length (3.793), suggesting more bacterial interactions and closer relationships among genera at this site. Meanwhile, the co-occurrence network for SS had the lowest modularity (0.379), reflecting significant differences in co-occurrence network topology for SS compared to the other three sites. Finally, the proportion of positive links (74.27%) was lowest in the PA co-occurrence network.

**Table 1:** Topological properties of the co-occurrence networks.

	Nodes	Links			Average degree	Modularity	Average clustering coefficient	Average path length
		Total	Positive	Negative				
TC	124	479	88.94%	11.06%	7.726	0.510	0.508	4.054
PA	170	583	74.27%	25.73%	6.859	0.576	0.445	3.954
SS	155	727	87.07%	12.93%	9.381	0.379	0.440	4.556
SA	242	1,278	87.17%	12.83%	10.562	0.606	0.541	3.793

TC, *Tamarix chinensis*; PA, *Phragmites australis*; SS, *Suaeda salsa*; SA, *Spartina alterniflora*.

### 3.6 Relationships between soil physicochemical properties and bacterial community structure

**Figure 6:**
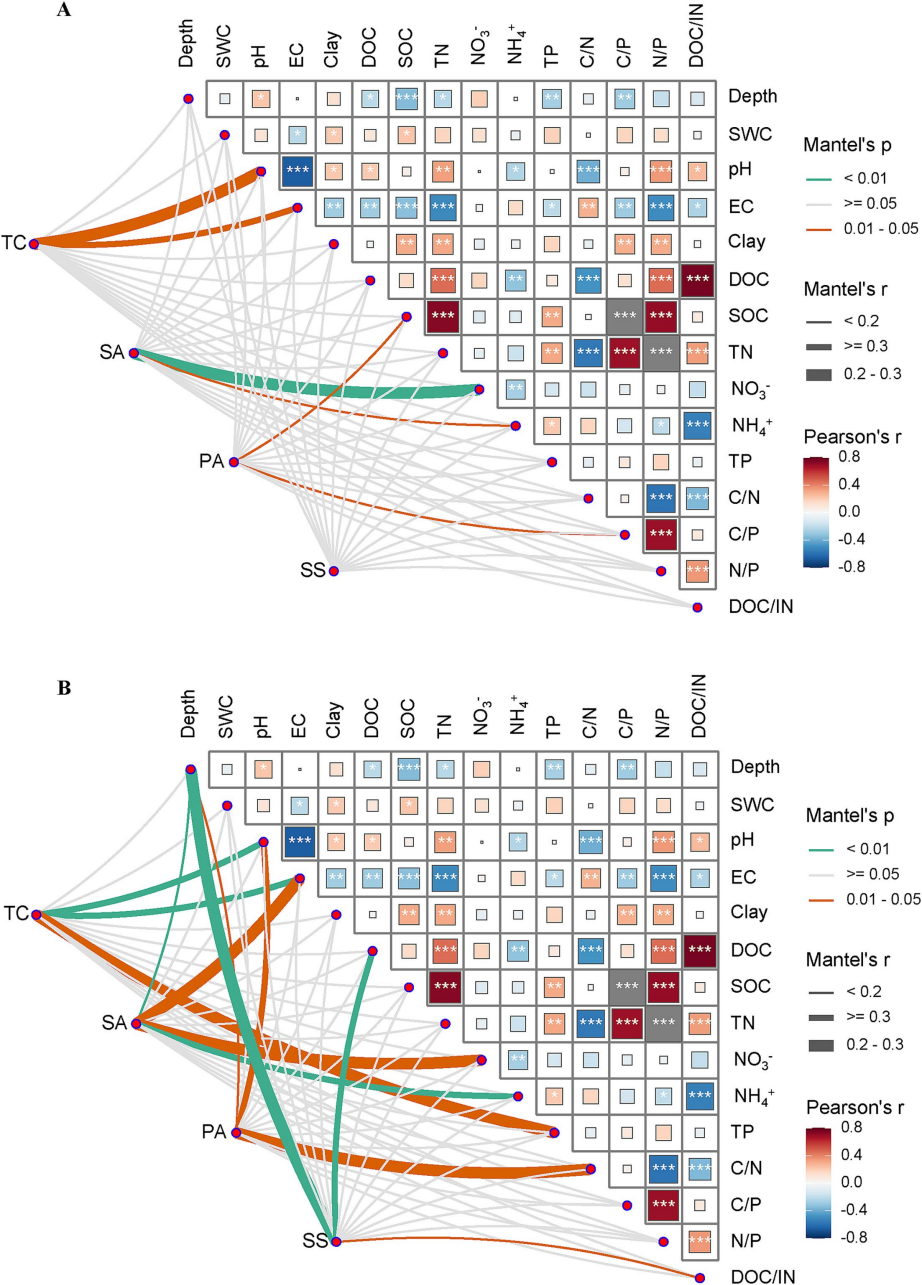
Associations between the α-diversity and composition of soil bacterial communities and various soil physicochemical properties across all study sites. Mantel tests examining connections between the α-diversity **(A)** and composition **(B)** of bacterial communities and various soil physicochemical properties at four study sites containing different plant species. DOC, dissolved organic carbon; EC, electrical conductivity; NH_4_^+^, ammonium; NO_3_^−^, nitrate; SOC, soil organic carbon; SWC, soil water content; TN, total nitrogen; TP, total phosphorus; TC, *Tamarix chinensis*; SA, *Spartina alterniflora;* PA, *Phragmites australis*; SS, *Suaeda salsa*.

The relationships between soil bacterial *α*-diversity or community composition and the soil physicochemical properties varied among sites (Figure 6). In TC, the bacterial community composition and Chao1 index were both significantly correlated with the soil EC and pH, while community composition was also correlated with TP. In SA, both the Chao1 index and community composition were correlated with NO_3_^−^ and NH_4_^+^, while community composition was also significantly correlated with the soil depth and EC. In PA, the Chao1 index and community composition were significantly correlated with aspects of C stoichiometry (C/P for the Chao1 index and C/N for community composition). By contrast, the Chao1 index of soil bacterial communities was not affected by any soil physicochemical properties in SS, whereas the composition of the SS communities was correlated with soil depth, DOC, and the DOC/IN ratio.

## 4 Discussion

### 4.1 Microbial community composition and diversity across study sites

The *α*-diversity of the soil bacterial community varied among study sites (Figure 2), with SA having higher α-diversity than the other three sites. *Spartina alterniflora,* an invasive plant with enhanced root exudates and litter inputs, greatly improved nutrient availability in the soil and thus supported greater bacterial α-diversity, in addition to affecting the composition and structure of the soil bacterial community (Song et al., 2020; Lin et al., 2022). Consistent with these results, Acidobacteria and Actinobacteria, widely recognized as *r*-strategists, were significantly more abundant than *K*-strategists (e.g., Bacteroidetes and Proteobacteria) in SA, supporting the finding that *S. alterniflora* affected the soil bacterial community by improving the nutrient content (Supplementary Table S2; Supplementary Figure S1). In addition, Mantel tests revealed that bacterial α-diversity in SA was correlated with the inorganic nitrogen content, which may be due to the fact that *S. alterniflora* significantly increased carbon resources in the soil (Figure 6A; Supplementary Table S1), leading to N-limitations for soil microorganisms.

In addition, substantial differences in bacterial community composition were observed among sites. Key bacterial genera involved in C-, N-, and S-cycling in coastal wetlands, including *Desulfuromonas*, *Marinobacterium*, *Nitrospira*, *Subgroup*_*10*, and *Woeseia*, were abundant at all four sites. However, the bacterial community composition varied across sites, especially for functions related to the N- and S-cycles. Using Mantel tests, differences in environmental drivers of community composition were found among sites. This suggests that variation in soil bacterial community composition was associated with site differences, including in local soil nutrient pools.

Among the bacterial taxa associated with N-cycling, the relative abundance of *Calorithrix* was relatively low in PA, while that of *Nitrospira* was elevated compared to other sites (Figures 3B,C). *Calorithrix* is associated with denitrification, while *Nitrospira* primarily participates in nitrification (Zhang X. et al., 2021; Daims and Wagner, 2018). Nitrification is regarded as a pivotal process in the production of soil N_2_O, potentially representing the primary mechanism (Lan et al., 2014). Here, the C/N ratio was the lowest among sites at PA, measuring 6.79: 1 (Supplementary Table S1), which is much lower than the average soil C/N ratio (10.1: 1) for coastal wetlands in China (Xia et al., 2023). Mantel test findings have shown that the soil bacterial composition at PA was correlated with the C/N ratio (Figure 6B). Sequently, Excess N may be removed by enhancing *Nitrospira*-based nitrification to reduce the adverse effects of a low C/N ratio (e.g., such as those caused by shifts in *Calorithrix* and *Nitrospira* abundance at PA). These results were confirmed by correlation test, which showed that higher TN and N/P and lower C/N at PA increased relative abundance of *Nitrospira* (Supplementary Figure S2).

Considering S-cycling, *Desulfurivibrio* and *Desulfuromonas* were most abundant in PA, whereas *SEEP-SRB1* was most common in SA (Figure 3B). *Desulfurivibrio*, *Desulfuromonas*, and *SEEP-SRB1* belong to the sulfate-reducing bacteria (SRB) (Petro et al., 2019; Yu et al., 2023) and are widely distributed in coastal wetlands, playing important roles in the S-cycle and participating in the release of wetland biosulfur gases and thereby mitigating sulfate stress in plants (Cui et al., 2017). In addition, sulfate can be reduced to sulfide via assimilatory sulfate reduction (ASR), an energy-consuming process, or it can be used as an electron acceptor in dissimilatory sulfate reduction (DSR) to generate energy (Yu et al., 2023). Previous studies have shown that *Desulfovibrio* and *SEEP-SRB1* participate in DSR and ASR, respectively (Yu et al., 2023; Murali et al., 2023). In our study, the enrichment of these three genera was primarily driven by soil nitrogen content. For instance, the high abundance of *Desulfurivibrio* and *Desulfuromonas* in PA sites was associated with elevated TN and NO₃^−^ levels, as well as higher N/P and lower C/N. Similarly, the high abundance of *SEEP-SRB1* was linked to elevated NO₃^−^ and reduced NH₄^+^ levels (Supplementary Figure S2). Interestingly, *Sulfurimonas* were also enriched in SA. *Sulfurimonas* are deep-sea chemoautotrophic bacteria capable of oxidizing sulfides (Wang et al., 2020). The co-occurrence of both sulfur-reducing and sulfur-oxidizing bacteria in SA is likely linked to the plant’s sulfur demands. Frequent tidal inundation in the low tidal zone, where *S. alterniflora* is located, promotes the accumulation of sulfur compounds, including acid-volatile sulfides, thereby supporting a higher abundance of sulfur-oxidizing bacteria such as *Sulfurimonas* (Li M. et al., 2021; Zheng et al., 2017). In contrast, other vegetation types like *S. salsa* and *T. chinensis*, located farther from the sea, experience less frequent tidal flooding and lower sulfur accumulation, which results in a lower abundance of sulfur-oxidizing bacteria. This dynamic balance between sulfur-reducing and sulfur-oxidizing bacteria in SA likely plays a key role in maintaining the sulfur cycle in its rhizosphere. Following *S. alterniflora* invasion and native plant succession, both plant species diversity and soil physicochemical properties were altered (Supplementary Table S1), resulting in changes in bacterial community composition among sites that were related to those in plant species composition.

### 4.2 Assembly of the bacterial community at different study sites

The null model results showed that deterministic and stochastic processes jointly drove bacterial community assembly at the four study sites, but stochastic processes dominated (Figure 4). The significance of stochastic processes for bacterial community assembly has been previously illustrated (Huo et al., 2023; Huang et al., 2022). Smaller organisms are more plastic in their metabolic abilities and have broader environmental tolerances; thus, bacterial communities are more affected by stochastic processes, according to the “size-plasticity” hypothesis (Wu et al., 2018; Zhu L. et al., 2024). However, it is worth noting that some studies have come to the opposite conclusion, showing that bacteria tend to have faster generation times and higher dispersal rates, which meaning they can respond to environmental changes more quickly and may be more susceptible to deterministic processes such as environmental filtering and/or biological interactions (Liu et al., 2023; Huo et al., 2023). The importance of deterministic versus stochastic processes may also depend on the specific microbial community and ecosystem being considered.

A conceptual model has been proposed to link the alternative hypotheses of ecosystem development to ecological assembly processes (Dini-Andreote et al., 2015). In this model, bacterial community composition is initially controlled by stochastic processes, while the role of deterministic selection increases with succession. As a relatively young wetland, the YRD continues to expand, forming new land (Qin et al., 2010). Relatively-infertile soils, repeated tidal action, and the lack of strong environmental filters allow stochastic processes to dominate microbial community assembly under these conditions. Environmental factors can then explain only a small fraction of the variation in bacterial community composition due to ongoing drift and dispersal (Fillinger et al., 2019; Danczak et al., 2018).

The strength of microbial responses to plant invasion is closely related to plant species identity, as well as to the duration and intensity of the invasion (Dostál et al., 2013; Zhang et al., 2020). Here, the amount of variation explained by stochastic processes was higher for SA versus the other sites, consistent with the findings of a previous study (Lin et al., 2022). The dominance of stochastic processes suggests that, when bacterial taxa have similar abilities to compete for common resources, taxa may coexist in highly overlapping ecological niches; this supports higher bacterial diversity as individual species are not eliminated by competition (Chase and Myers, 2011; Jiao et al., 2020). The assembly of high-diversity bacterial communities is mainly mediated by stochastic processes, while deterministic processes are more important when bacterial diversity is low, likely due to the reduction in specialized functions correlated with specific bacterial taxa (Xun et al., 2019). At SA, greater bacterial abundance and diversity enhanced the environmental tolerance and metabolic plasticity of the bacterial community, thus reducing the influence of environmental factors and increasing the importance of stochastic processes (Chen and Wen, 2021). The importance of stochasticity in bacterial community assembly may also increase with resource availability (Chase, 2010; Zheng et al., 2021). Thus, the higher nutrient availability and bacterial diversity associated with *S. alterniflora* invasion might favor stochastic processes in bacterial community assembly in coastal wetland soils.

### 4.3 Microbial co-occurrence networks for different study sites

Network analysis can be applied to identify species co-occurrence patterns and potential interactions in complex biotic communities (Faust and Raes, 2012; Zhu L. et al., 2024). Here, the bacterial co-occurrence network for SA had more nodes and edges, higher modularity values, and shorter average path lengths than the networks created for the other sites (Table 1), indicating greater complexity. This is because high modularity values indicate that while the nodes within a module are highly connected, effects on the rest of the network are reduced; this helps to maintain a community of complex interactions (Qiao et al., 2024). In addition, species in a module may be ecologically similar with overlapping niches (Huo et al., 2023). Niche overlap induces complex interactions in the community, explaining why the co-occurrence network of SA had more nodes and edges. The average path length is defined as the average number of steps along the shortest path between each pair of nodes, and represents a measure of network efficiency (Zhou et al., 2010). The low average path length in the SA network indicates faster information transmission within the network, allowing the microbial community to respond more quickly to environmental change (Zhou et al., 2010). *S. alterniflora*, located in the low tidal zone, is subject to frequent tidal inundation, which results in rapid transitions between aerobic and anaerobic conditions in the soil (Gao et al., 2018; Wei et al., 2020). Additionally, the high levels of sulfur accumulation associated with *S. alterniflora* invasion may cause stress to the soil bacterial community (Li M. et al., 2021). Consequently, faster information transmission within bacterial networks could enhance soil bacterial survival, enabling the bacterial community to better cope with stressful environmental conditions.

Positive interactions between microorganisms include cooperation, commensalism, and mutualism, while negative interactions include competition, parasitism, and predation (Zhu P. et al., 2024). The larger proportion of positive versus negative correlations in all four co-occurrence networks indicates the presence of cooperative or syntrophic interactions, suggesting that mutualistic interactions were common among soil bacterial taxa (Gao et al., 2021; Li C. et al., 2021). The coastal wetlands of YRD are characterized by relatively infertile soils and frequent tidal action, creating stressful conditions that promote mutualistic interactions within soil bacterial communities (Hernandez et al., 2021). Nevertheless, the PA network exhibited a greater number of negative interactions than the other three sites, suggesting a more competitive microbial environment. The supratidal zone and late successional plant species in PA contributed to improvements in soil nutrient availability, as evidenced by the relatively high SOC, TN, and TP, as well as the low C/N ratio. These factors likely contributed to the development of more competitive associations within the bacterial community.

## 5 Conclusion

In the YRD coastal wetlands, plant species identity was more influential than soil depth for shaping the soil bacterial community. However, it should be noted that other environmental factors, such as tidal inundation, also significantly contribute to microbial community composition. *Spartina alterniflora* invasion strongly impacted the soil microbial community, altering its structure and increasing bacterial *α*-diversity. Additionally stochastic processes largely drove bacterial community assembly, with *S. alterniflora* invasion further enhancing their dominance. The co-occurrence networks at the study sites primarily featured positive interactions, with *S. alterniflora* exhibiting higher complexity and modularity. These findings underscore the importance of plant communities in shaping soil bacterial diversity while also considering the influence of environmental factors like tidal flooding.

## Data Availability

The 16S rRNA sequencing data presented in this study are deposited in the NCBI SRA repository, BioProject accession number PRJNA1217841.

## Footnotes

^1^ https://cloudoebiotechcom/task/
