# Supplementary material for: Spartina alterniflora invasion significantly alters the assembly and structure of soil bacterial communities in the Yellow River Delta
Source: Front Microbiol. 2025 Feb 12;16:1525632. doi: 10.3389/fmicb.2025.1525632 (PMC11861095; doi:10.3389/fmicb.2025.1525632)
Supplement: Supplementary file 1 [file Data_Sheet_1.docx]

Supplementary Materials

# Supplementary Figures and Tables

## Supplementary Figures


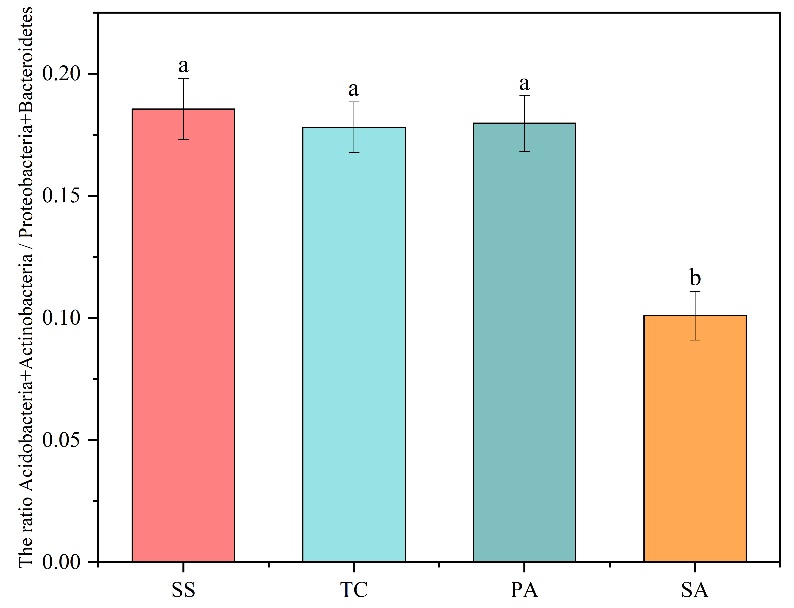


**Fig. S1. The ratio of *K*-strategists to *r*-strategists by study site.**


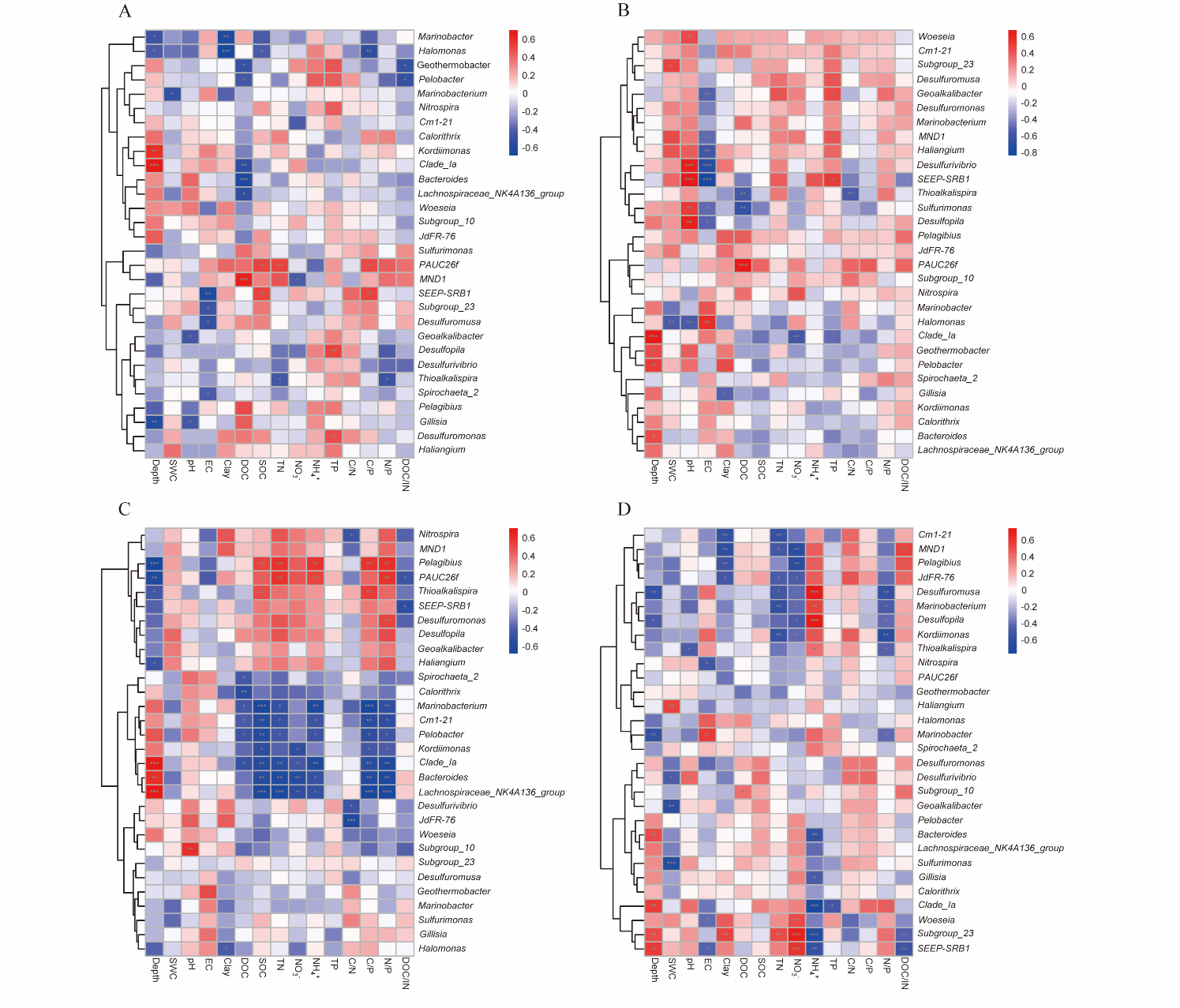


**Fig. S2. Associations between the top 30 genera in all soil bacterial communities and various soil physicochemical properties across the four study sites.** **The correlations were calculated using Spearman's rank correlation method. A: *Suaeda salsa* (SS); B: *Tamarix chinensis* (TC); C: *Phragmites australis* (PA); D: *Spartina alterniflora* (SA).**

## Supplementary Tables

**Table S1. Soil physicochemical properties at four study sites characterized by different plant species.**

| Community type | Soil depth | C/N | DOC (mg kg^-1^) | EC (mS cm^-1^) | NH_4_^+^ – N (mg kg^-1^) | NO_3_^-^ – N (mg kg^-1^) | pH | SOC (g kg^-1^) | SWC (%) | TN (g kg^-1^) | TP (g kg^-1^) |
| --- | --- | --- | --- | --- | --- | --- | --- | --- | --- | --- | --- |
| *Suaeda salsa* | 0 – 10 cm | 20.78 ± 10.69^Aa^ | 23.95 ± 5.19^Ab^ | 2.14 ± 0.45^Aab^ | 5.75 ± 5.44^ABa^ | 1.45 ± 0.24^Aa^ | 9.12 ± 0.25^Aa^ | 3.50 ± 0.94^Ac^ | 30.10 ± 2.48^Aa^ | 0.20 ± 0.10^Ab^ | 0.50 ± 0.04^Aa^ |
|  | 10 – 20 cm | 15.37 ± 6.48^Aa^ | 17.96 ± 3.22^ABb^ | 2.18 ± 0.85^Aa^ | 5.21 ± 7.32^ABa^ | 1.59 ± 0.95^Ab^ | 9.27 ± 0.16^Ab^ | 3.00 ± 1.10^Abc^ | 28.02 ± 2.24^Aa^ | 0.21 ± 0.09^Ab^ | 0.51 ± 0.08^Aa^ |
|  | 20 – 30 cm | 16.72 ± 7.96^Aa^ | 16.44 ± 3.87^Bb^ | 2.25 ± 0.50^Aa^ | 19.70 ± 9.33^Aa^ | 1.58 ± 0.14^Ab^ | 9.14 ± 0.18^Ab^ | 3.10 ± 0.95^Ab^ | 27.88 ± 2.45^Aa^ | 0.22 ± 0.12^Ab^ | 0.50 ± 0.03^Aa^ |
|  | 30 – 40 cm | 13.83 ± 4.07^Aab^ | 15.17 ± 2.36^Bb^ | 2.25 ± 0.40^Aab^ | 0.75 ± 0.66^Bab^ | 2.98 ± 1.43^Aab^ | 9.32 ± 0.19^Ab^ | 3.28 ± 0.88^Aa^ | 28.74 ± 1.75^Aa^ | 0.25 ± 0.10^Ab^ | 0.46 ± 0.05^Aa^ |
| *Tamarix chinensis* | 0 – 10 cm | 16.83 ± 2.50^Aa^ | 20.00 ± 2.62^Ab^ | 2.39 ± 1.37^Aab^ | 3.08 ± 2.85^Aa^ | 2.83 ± 1.91^Aa^ | 9.11 ± 0.52^Aa^ | 5.73 ± 1.16^Ab^ | 32.46 ± 5.64^Aa^ | 0.34 ± 0.03^Ab^ | 0.53 ± 0.03^Aa^ |
|  | 10 – 20 cm | 14.09 ± 5.43^Aa^ | 20.80 ± 5.39^Ab^ | 1.86 ± 0.59^Aa^ | 4.03 ± 6.09^Aa^ | 2.83 ± 1.29^Aab^ | 9.34 ± 0.37^Aab^ | 3.74 ± 0.54^Bab^ | 31.37 ± 3.37^Aa^ | 0.29 ± 0.09^Ab^ | 0.50 ± 0.02^Aa^ |
|  | 20 – 30 cm | 15.95 ± 5.67^Aab^ | 18.17 ± 6.60^Ab^ | 2.17 ± 1.02^Aa^ | 6.38 ± 6.79^Aab^ | 2.04 ± 0.47^Aab^ | 9.42 ± 0.27^Aab^ | 3.37 ± 0.67^Bb^ | 31.03 ± 1.75^Aa^ | 0.24 ± 0.10^Ab^ | 0.49 ± 0.04^Aa^ |
|  | 30 – 40 cm | 15.02 ± 4.45^Aa^ | 20.65 ± 10.51^Aab^ | 2.59 ± 1.30^Aa^ | 2.33 ± 3.00^Aab^ | 1.96 ± 0.39^Ab^ | 9.44 ± 0.25^Aab^ | 3.23 ± 0.80^Ba^ | 31.94 ± 3.31^Aa^ | 0.25 ± 0.15^Ab^ | 0.48 ± 0.05^Aa^ |
| *Phragmites australis* | 0 – 10 cm | 6.72 ± 1.12^Ab^ | 50.24 ± 20.25^Aa^ | 1.13 ± 0.31^Ab^ | 2.38 ± 0.61^Aa^ | 1.66 ± 0.14^Aa^ | 9.52 ± 0.19^Aa^ | 8.49 ± 1.29^Aa^ | 31.24 ± 5.23^Aa^ | 1.28 ± 0.18^Aa^ | 0.54 ± 0.05^Aa^ |
|  | 10 – 20 cm | 6.19 ± 0.95^Ab^ | 39.22 ± 15.50^Aab^ | 0.94 ± 0.17^Ab^ | 1.68 ± 0.39^ABa^ | 2.21 ± 1.13^Aab^ | 9.70 ± 0.18^Aa^ | 4.72 ± 0.87^BCa^ | 31.32 ± 2.23^Aa^ | 0.78 ± 0.18^Ba^ | 0.52 ± 0.05^Aa^ |
|  | 20 – 30 cm | 7.48 ± 1.53^Ab^ | 41.83 ± 12.20^Aa^ | 1.08 ± 0.22^Ab^ | 1.66 ± 0.44^Bab^ | 2.27 ± 1.31^Aab^ | 9.59 ± 0.25^Aa^ | 5.35 ± 0.96^Ba^ | 29.18 ± 3.30^Aa^ | 0.74 ± 0.18^Ba^ | 0.51 ± 0.04^Aa^ |
|  | 30 – 40 cm | 6.76 ± 2.84^Ac^ | 30.52 ± 6.69^Aa^ | 1.26 ± 0.28^Ab^ | 1.04 ± 0.22^Ba^ | 1.61 ± 0.80^Ab^ | 9.73 ± 0.06^Aa^ | 3.28 ± 0.53^Ca^ | 29.84 ± 4.87^Aa^ | 0.53 ± 0.16^Ba^ | 0.51 ± 0.07^Aa^ |
| *Spartina alterniflora* | 0 – 10 cm | 7.94 ± 3.41^Ab^ | 44.79 ± 13.48^Aa^ | 2.47 ± 0.62^Aa^ | 0.82 ± 0.37^Aa^ | 2.74 ± 1.61^Aa^ | 9.42 ± 0.14^Aa^ | 2.57 ± 0.87^Ac^ | 29.91 ± 3.20^Aa^ | 0.35 ± 0.11^Ab^ | 0.49 ± 0.04^Aa^ |
|  | 10 – 20 cm | 7.44 ± 2.45^Ab^ | 59.36 ± 31.71^Aa^ | 1.87 ± 0.27^Aa^ | 0.68 ± 0.21^Aa^ | 3.32 ± 0.55^Aa^ | 9.52 ± 0.11^Aab^ | 2.32 ± 0.67^Ac^ | 29.26 ± 2.35^Aa^ | 0.32 ± 0.04^Ab^ | 0.47 ± 0.06^Aa^ |
|  | 20 – 30 cm | 9.15 ± 3.60^Aab^ | 41.36 ± 13.73^Aa^ | 1.99 ± 0.25^Aab^ | 0.43 ± 0.38^ABb^ | 5.35 ± 2.23^Aa^ | 9.45 ± 0.22^Aab^ | 3.07 ± 0.60^Ab^ | 30.55 ± 3.98^Aa^ | 0.36 ± 0.08^Ab^ | 0.48 ± 0.07^Aa^ |
|  | 30 – 40 cm | 8.76 ± 2.19^Abc^ | 33.73 ± 12.06^Aa^ | 1.85 ± 0.38^Aab^ | 0.11 ± 0.24^Bb^ | 5.60 ± 2.56^Aa^ | 9.52 ± 0.06^Aab^ | 3.09 ± 0.74^Aa^ | 30.59 ± 5.26^Aa^ | 0.35 ± 0.04^Aab^ | 0.44 ± 0.02^Aa^ |

Abbreviations: DOC, dissolved organic carbon; EC, electrical conductivity; NH_4_^+^ – N, ammonium; NO_3_^-^ – N, nitrate; SOC, soil organic carbon; SWC, soil water content; TN, total nitrogen content; TP, total phosphorus content. Values represent averages ± the standard deviation. Different uppercase letters (e.g., “A”, “B”, etc.) indicate significant differences (ANOVA, *p* < 0.05) between soil depths. Different lowercase letters (e.g., “a”, “b”, etc.) indicate significant differences (ANOVA, *p* < 0.05) between sites.

**Table S2. One-way ANOVAs comparing the relative abundance of major bacterial phyla among study sites (i.e., plant communities) and soil depths.**

| Phylum | Study site | Soil depth | Study site  * soil depth |
| --- | --- | --- | --- |
| Proteobacteria | *p*>0.05 | *p*>0.05 | *p*>0.05 |
| Bacteroidetes | *p*>0.05 | *p*>0.05 | *p*>0.05 |
| Acidobacteria | ***p*<0.001** | ***p*=0.001** | *p*>0.05 |
| Actinobacteria | ***p*<0.001** | *p*>0.05 | *p*>0.05 |
| Gemmatimonadetes | ***p*=0.003** | *p*>0.05 | *p*>0.05 |
| Firmicutes | *p*>0.05 | *p*>0.05 | *p*>0.05 |
| Nitrospirae | ***p*<0.001** | *p*>0.05 | *p*>0.05 |
| Epsilonbacteraeota | *p*>0.05 | *p*>0.05 | *p*>0.05 |
| Patescibacteria | ***p*=0.004** | *p*>0.05 | *p*>0.05 |
| Calditrichaeota | *p*>0.05 | ***p*=0.035** | *p*>0.05 |
